# Near Infrared Spectroscopy Measurements of Mitochondrial Capacity Using Partial Recovery Curves

**DOI:** 10.3389/fphys.2020.00111

**Published:** 2020-02-14

**Authors:** Maxwell D. Sumner, Samuel Beard, Elizabeth K. Pryor, Indrajit Das, Kevin K. McCully

**Affiliations:** Non-Invasive Exercise Muscle Physiology Lab, Department of Kinesiology, University of Georgia, Athens, GA, United States

**Keywords:** NIRS, skeletal muscle, muscle metabolism, electrical stimulation, human subjects

## Abstract

**Background:**

Near-infrared spectroscopy (NIRS) has been used to measure muscle mitochondrial capacity (mVO_2_max) as the recovery rate constant of muscle metabolism after exercise. The current method requires as many as 50 short ischemic occlusions to generate two recovery rate constants.

**Purpose:**

To determine the validity and repeatability of using a 6-occlusion protocol versus one with 22 occlusions to measure muscle mitochondrial capacity. The order effect of performing multiple Mito6 test was also evaluated.

**Method:**

In two independent data sets (bicep *n* = 7, forearm A *n* = 23), recovery curves were analyzed independently using both the 6 and 22 occlusion methods. A third data set (forearm B *n* = 16) was generated on the forearm muscles of healthy subjects using four 6-occlusion tests performed in succession. Recovery rate constants were generated using a MATLAB routine.

**Results:**

When calculated from the same data set, the recovery rate constants were not significantly different between the 22 occlusion and 6 occlusion methods for the bicep (1.43 ± 0.33 min^–1^, 1.43 ± 0.35 min^–1^, *p* = 0.81) and the forearm A (1.97 ± 0.40 min^–1^, 1.97 ± 0.43 min^–1^, *p* = 0.90). Equivalence testing showed that the mean difference was not different than zero and the 90% confidence intervals were within 5% of the average rate constant. This was true for the Mito6 and the Mito5^∗^ approaches. Bland–Altman analysis showed a slope of 0.21 min^–1^ and an r of 0.045 for the bicep dataset and a slope of −0.01 min^–1^ and an *r* of 0.045 for the forearm A dataset. When performing the four 6-occlusion tests; recovery rate constants showed no order effects (1.50 ± 0.51 min^–1^, 1.42 ± 0.54 min^–1^, 1.26 ± 0.41 min^–1^, 1.29 ± 0.47 min^–1^, *P* > 0.05).

**Conclusion:**

The Mito6 analysis is a valid and repeatable approach to measure mitochondrial capacity. The Mito6 protocol used fewer ischemic occlusion periods and multiple tests could be performed in succession in less time, increasing the practicality of the NIRS mitochondrial capacity test. There were no order effects for the rate constants of four repeated 6-occlusion tests of mitochondrial capacity, supporting the use of multiple tests to improve accuracy.

## Introduction

Near Infrared Spectroscopy (NIRS) has been used as a non-invasive approach to measuring muscle oxygen levels (Grassi and Quaresima, 2016; Hamaoka and McCully, 2019). NIRS protocols have been developed to measure muscle mitochondria capacity (mVO_2_max) as the rate constant of recovery of muscle metabolism during the transition from exercise to rest (Hamaoka et al., 1999; Motobe et al., 2004; Ryan et al., 2012; Adami and Rossiter, 2018). The NIRS approach has shown agreement with similar measurements of phosphocreatine recovery rates (Meyer, 1988; Ryan et al., 2013b), and with mitochondrial respiration rates from tissue biopsies (Ryan et al., 2014a). The method has also been used to measure muscle mitochondrial capacity in clinical populations (Willingham and McCully, 2017): such as people with spinal cord injuries (Erickson et al., 2013), cystic fibrosis (Erickson et al., 2015), multiple sclerosis (Harp et al., 2016), and amyotrophic lateral sclerosis (Ryan et al., 2014b).

There are several important limiting factors to the use of NIRS to measure muscle mitochondrial capacity. One factor is the need to use repeated short duration arterial occlusions after exercise (Hamaoka et al., 1999). These occlusions are needed to obtain the measurements of metabolic rate required to determine the rate constant of recovery of muscle metabolism. Typically, 18–26 ischemic occlusions are needed to accurately fit the metabolic rates to a mono-exponential curve approaching a steady baseline for a single rate constant to be generated (Sako et al., 2001; Motobe et al., 2004). As 2–3 trials are typically performed to obtain an accurate assessment of the recovery rate constant (Adami and Rossiter, 2018; Lagerwaard et al., 2019), 36–78 short cuff occlusions are required over 30–45 min (Willingham and McCully, 2017). This large number of cuffs can be difficult to tolerate, especially in at-risk and elderly populations.

A second limiting factor is the requirement for a blood volume correction factor (Ryan et al., 2012) in order to correct for changes in light absorption on the metabolic rate measurements when the ischemic cuff inflates. The magnitude of this correction remains constant throughout the test, such that the influence of the correction is greatest on the measurements of metabolic rate at the end of the recovery period when metabolic rate is low (Ryan et al., 2012). Therefore, the necessity for accurate blood volume corrections are less important for the initial recovery time points, with high metabolic rates, than for later recovery time points, with smaller metabolic rates, as these points are less affected by the standard correction. Thus, an analysis approach that makes use of only the higher metabolic rate data could improve the accuracy of curve fitting a rate constant.

The aim of this study is to evaluate an abbreviated recovery test protocol using 6 cuff occlusions (Mito6) for measuring muscle mitochondrial capacity using NIRS. It is hypothesized that using incomplete recovery curves (Mito6) to produce rate constants is a valid and repeatable substitute for the use of complete recovery curves (Mito22) to produce rate constants. Additionally, we hypothesize that if four Mito6 protocols are performed sequentially, there will not be an order effect seen in the rate constants. By shortening the time and effort required to perform a mitochondrial test, the new abbreviated Mito6 protocol would have advantages over the currently used Mito22 protocol. The use of analysis approaches that do not require a full metabolic recovery for each successive test could be performed in a shorter period of time, with data that is less influenced by the need for a correction factor.

## Materials and Methods

### Participants

This study made use of three independent data sets (subjects: bicep *n* = 7, forearm A *n* = 23, and forearm B *n* = 16). Subject characteristics are shown in Table 1. All subjects were healthy. Multiple tests were made on each subject such that the total number of tests in each group were the following: bicep = 47; forearm A = 39; forearm B = 16. Comparisons were made between individual test in each group and in some cases the average values for each subject within a group. All studies were conducted with approval of the Institutional Review Board at the University of Georgia (Athens, GA, United States), and all of the subjects gave written, informed consent before testing.

**TABLE 1:** Subject characteristics.

Variable	Bicep		Forearm A		Forearm B	
			
	Male	Female	Male	Female	Male	Female
Subjects (#)	2	5	10	13	6	10
Tests (#)	18	29	17	22	6	10
Age (years)	20 ± 0	20 ± 0	28 ± 6	23 ± 3	20 ± 9	21 ± 8
Height (m)	1.71 ± 0.90	1.68 ± 1.16	1.80 ± 0.57	1.65 ± 0.66	1.55 ± 0.6	1.64 ± 0.5
Weight (kg)	65.1 ± 9.3	61.1 ± 8.8	86.0 ± 10.8	64.5 ± 7.0	66.3 ± 29.0	53.3 ± 17.7
BMI (m^2^/kg)	22.1 ± 0.9	21.6 ± 2.5	26.5 ± 3.2	23.7 ± 2.6	20.2 ± 8.6	19.8 ± 6.6
ATT (cm)	0.25 ± 0.1	0.17 ± 0.9	0.36 ± 0.08	0.54 ± 0.16	0.31 ± 0.2	0.23 ± 0.1

*Values are means ± SD. BMI, body mass index; ATT, adipose tissue thickness.*

### Experimental Procedure

Near-infrared spectroscopy was used to measure muscle mitochondrial capacity (Willingham and McCully, 2017). Briefly, continuous wavelength NIRS was used to measure oxygen levels in skeletal muscle, short duration ischemic cuff periods were used to determine muscle metabolism, and a series of short ischemic cuff durations after electrical stimulation were used to measure the rate constant of recovery from a high to a low metabolic rate. The rate constant of the recovery from high to low metabolic rate was used as an index of muscle mitochondrial capacity. A continuous wavelength NIRS device (Portamon, Artinis Medical Systems, Elst, Netherlands) with 4 cm separation distance was placed over the muscle of interest. Stimulation electrodes were placed on either side of the muscle of interest (proximal and distal for the forearm muscles, and medial and lateral for the bicep muscles). The muscle was activated using electrical twitch stimulation at either 4 or 6 Hz for 30 s at a current level that produced vigorous but submaximal contractions (Ryan et al., 2013a). A 5 cm wide blood pressure cuff was placed proximal to the muscle and connected to a rapid cuff inflation device and air source. Ischemic periods were produced with rapid (approximately 1 s) cuff inflations to greater than 200 mmHg.

#### Establishing Validity and Repeatability

**FIGURE 1:**
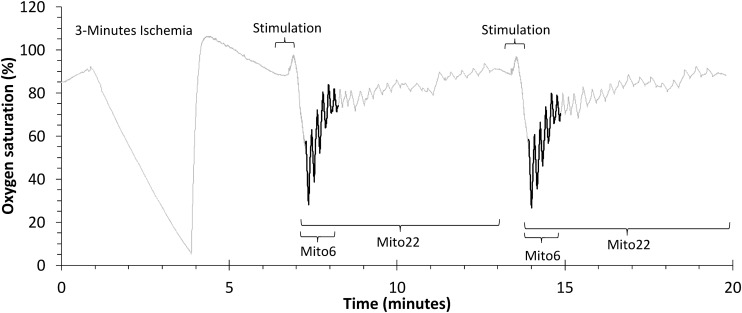
A representative file of oxygen saturation during a mitochondrial capacity test in the bicep muscle group. The Mito22 analysis uses all of the ischemic cuffs in the test. The Mito6 analysis uses the first 6 ischemic cuffs (in bold) and a final resting cuff.

The first portion of this study involved comparisons between two different methods of data analysis. The first method, Mito22, calculated recovery rate constants based on a full recovery curve, using values from 22 ischemic cuff periods. The second method, Mito6, calculated recovery rate constants using the first six recovery values and a final, delayed, end-stimulation value. A representative example of the protocol used for the 22 and 6 cuff approaches are shown in Figure 1. Both methods were performed simultaneously to independently measure recovery values based on identical data values. Comparisons of both approaches were performed on the bicep and forearm A datasets.

##### Approach 1 – Mito22

The values recorded were then entered into a custom-written routine in MatLab v 9.2.0.556344 (Mathworks, Natick, MA, United States) for analysis. A recovery rate constant was calculated using a standard mitochondrial capacity analysis (Ryan et al., 2014b). Data from all 22 ischemic cuffs was fit to an exponential equation y(t) = End − Δ × e^(−kt)^. Y(*t*) is the metabolic rate at time *t*, End is the metabolic rate at full recovery, Δ is the difference in metabolic rate from high metabolic activity to full recovery. *k i*s the recovery rate constant used as an index of muscle oxidative capacity.

##### Approach 2 – mito6

The first six ischemic cuff slopes and the last cuff slope were used to match to a mono-exponential curve to a flat baseline. The decision was made to use as few cuffs as possible. Six ischemic cuffs were chosen because with a duration of 5 s on and 5 s off, this number of cuffs would include the steepest part of the recover curve in most human subjects. Using the same exponential equation as for the Mito22 analysis, the end and delta values were determined from the initial and final ischemic cuff slopes. Then a series of equations were generated using a wide range of rate constants. For each equation, a combined residual was determined using the first six data points and the final resting data point. The curve with the lowest combined residual values was selected as the Mito6 recovery rate constant. Additionally, the influence on the mitochondrial rate constants determined from the Mito6 test of using either an initial resting or a final resting cuff, as well as the elimination of the point with the highest residual value (Mito5^∗^) was evaluated.

Multiple approaches to an abridged mitochondrial capacity test analysis were attempted. These attempts included systematically eliminating the point with the highest residual value and refitting the mono-exponential curves with the remaining five values (Mito5^∗^). Non-parametric correlations using Spearman’s rho were performed comparing both the Mito6 and Mito5^∗^ methods. A separate approach that was attempted included using an initial resting value rather than a final resting value to establish the mono-exponential curves. Each of these attempts was ineffective in establishing an abridged mitochondrial capacity test.

#### Evaluation of Order Effects in Sequential Mito6 Protocols

**FIGURE 2:**
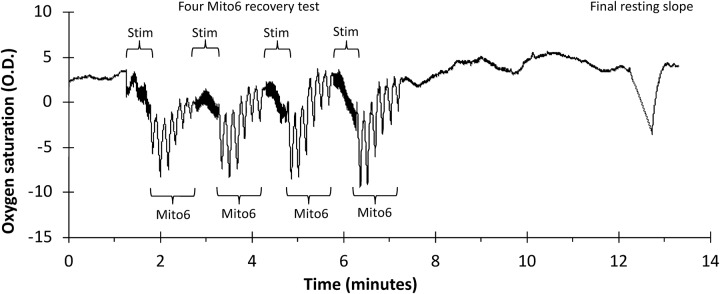
A representative file of oxygen saturation using a Mito6 protocol in the forearm muscle group. Mito6 analysis uses 6 ischemic cuffs and a final resting cuff.

In the second portion of this study, data was collected from the forearm muscles (forearm B) of young adult subjects (Table 1) in which only the Mito6 protocol was conducted sequentially four times in order to determine if order effects were present in repeated use of the Mito6 protocol. The protocol is shown in Figure 2. A NIRS device (Portamon, Artinis Medical Systems, Elst, Netherlands) with 4 cm separation distance was placed on the medial forearm flexor muscles of each participant’s right arm. Two electrical stimulation electrodes were placed adjacent to the NIRS device. The participant reclined in a supine position throughout the duration of the test. The protocol for each assessment began with a period of rest (*t* = 5 min), followed by a stimulation check (stim check; 30 s cuff inflation, 30 s rest, 30 s electrical stimulation, and then a final 30 s cuff inflation) to ensure the electrodes were working properly and stimulation was strong enough to activate the muscle adequately. A set of four Mito6 tests were then performed sequentially. Each test consisted of 30 s of electrical stimulation followed by six ischemic arterial occlusions (cuff inflations). Each cuff inflation lasted 5 s, and 5 s recovery. After the fourth Mito6 test, a 5-min rest period occurred to allow recovery. A final 30 s cuff inflation was then initiated to determine end metabolic rate. Following the NIRS measurements, subcutaneous adipose tissue thickness superficial to the right forearm flexors was measured, using ultrasound imaging.

### Data Analysis and Statistics

The measured rate constants of both approaches were compared through correlation analysis and linear regression. The final Mito22 and Mito6 analysis protocols of averaged multiple trials were compared to determine accuracy and usability. For comparisons of the averaged multiple trial values collected, Pearson correlations were performed for the 6-cuff and 22-cuff analyses. Mito6 Analysis was performed with all six points (and the last rest value) and with one of the first six points removed (Mito5^∗^). To establish validity and repeatability, Equivalence testing was performed between the Mito22 and the Mito6 and Mito5^∗^ analyses for the biceps and forearm A data sets (Lakens, 2017). This testing evaluated the mean difference between the two analyses (Mito22 and Mito6/Mito5^∗^) and determined the 90% confidence interval for the mean difference and the equivalence bounds which was assumed to be 5% of the mean rate constant values. This approach provides similar analysis outcomes to that for Bland–Altman analyses (Altman and Bland, 1983; Dogan, 2018). Bland–Altman analysis was also performed and the slope and *R*^2^-values for the rate constants of the data: (Mito22-Mito6)/(Mito22 + Mito6)/2, was performed to evaluate potential changes in agreement with the rate constants and magnitude of the rate constant (Altman and Bland, 1983). Interclass correlation coefficients (ICC) values were also generated using a two-way mixed effects model using average values where people effects were random and measures effects were fixed. The ICC values generated refer to within subject comparisons. Paired, two-tailed *t*-test were also performed on the multiple trial averages to generate *p*-values between testing methods.

Mito22 to Mito6 comparisons also included arbitrarily separating data by the *R*^2^-value for the Mito22 fit of the exponential curve. Data was separated into two categories based upon the midpoint of the range of the measured *R*^2^-value. Data that had *R*^2^-values above the midpoint value were labeled “Above Midpoint” and data with *R*^2^-value below the midpoint were labeled ‘Below Midpoint.’ Comparisons were made between the Mito22 and Mito6 data using all the individual mitochondrial tests (often 2 per test session) and for averaged Mito22 and Mito6 data, where multiple tests in the same experiment were averaged. These comparisons were completed through correlation analysis and linear regression. When 4 mito6 tests were performed in succession, evaluation of the effect of testing order on both the mitochondrial rate constant and the initial metabolic rate (first measurement after the end of stimulation) were made.

## Results

### Rate Constant Comparisons Between Mito6 and Mito22

When calculated from the same data set, the recovery rate constants were not significantly different between the 22 occlusion and 6 occlusion methods for the bicep (1.43 ± 0.33 min^–1^, 1.43 ± 0.35 min^–1^, *p* = 0.81) and the forearm A (1.97 ± 0.40 min^–1^, 1.97 ± 0.43 min^–1^, *p* = 0.90).

**FIGURE 3:**
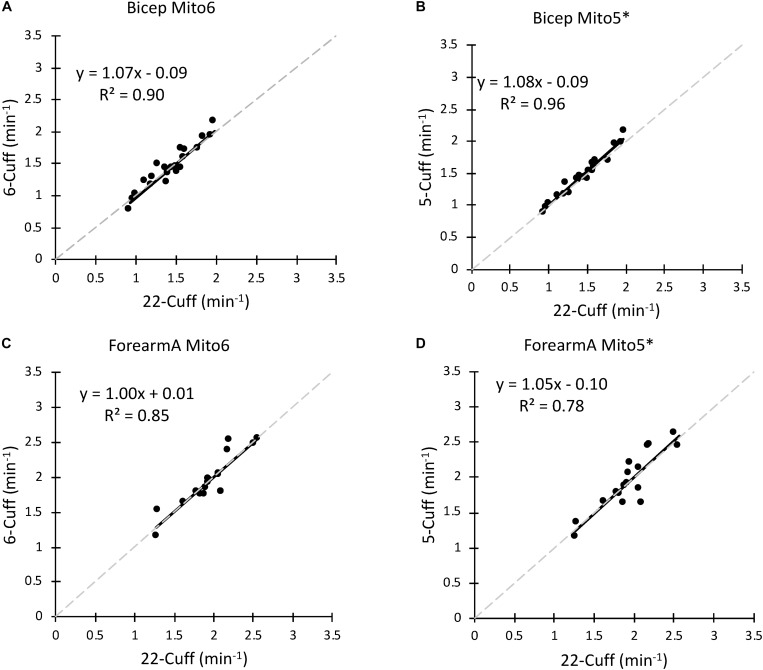
**(A)** A comparison of the Mito22 and Mito6 analysis protocols using bicep muscle data. **(B)** A comparison of the Mito22 and Mito5^∗^ analysis protocols using bicep muscle data. **(C)** A comparison of the Mito22 and Mito6 analysis protocols using forearm A muscle data. **(D)** A comparison of the Mito22 and Mito5^∗^ analysis protocols using forearm A muscle data. For all figures, data is an average of two trials on the same subject. The gray dashed line represents the line of identity in all graphs.

Data analysis was performed with all points (Mito6) and with the point with the highest residual removed (Mito5^∗^) compared to a Mito22 analysis. Comparisons for the bicep and forearm A data sets between the Mito6 and Mito5^∗^ analyses are shown in Figure 3. For the bicep data set, the removal of a point did increase the correlation coefficient (0.90 for Mito6 and 0.96 for Mito5^∗^). For the Forearm A data set, the removal of a point did not increase the correlation coefficient (0.85 for Mito6 and 0.78 for Mito5^∗^).

**FIGURE 4:**
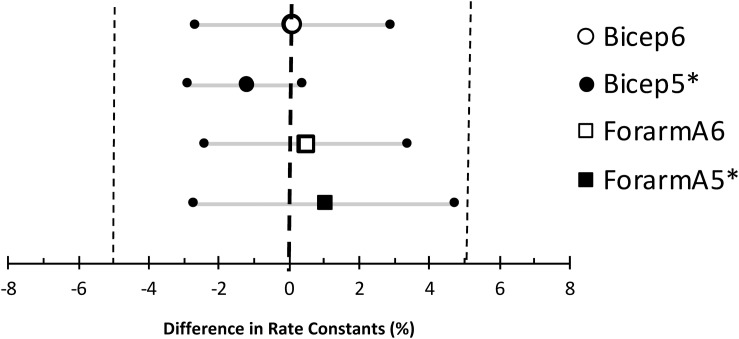
Equivalence testing comparing the Mito 22 results to the Mito6 and Mito5^∗^ results for the bicep and forearm A data sets. The large symbols are the mean differences between the measurements, the small symbols with the gray lines are the 90% confidence intervals, and the small vertical dotted lines are the range of minimal significance difference (5%).

**FIGURE 5:**
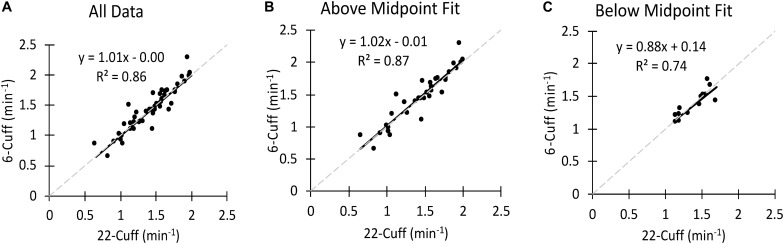
**(A)** A comparison of the Mito22 and Mito6 analysis protocols using bicep muscle data. **(B)** A comparison of the Mito22 and Mito6 analysis protocols using Above Midpoint Fit Biceps data. **(C)** A comparison of the Mito22 and Mito6 analysis protocols using Below Midpoint Fit Biceps data. For all figures the data include separate tests (two tests per subject). The gray dashed line represents the line of identity in all graphs.

To establish validity and repeatability, equivalence testing (Figure 4) showed the data for both the bicep and forearm A data sets to be statistically equivalent and not different. This was true for the Mito6 and the Mito5^∗^ approaches. These conclusions were based on that the differences between these values and the Mito22 values were not different than zero, and the 90% confidence interval for the mean difference was within 5% of the mean value. The Bland–Altman analysis showed that within subject repeatability was independent of the size of the rate constant, as the slope was 0.21 min^–1^ and r was 0.045 for the bicep dataset and the slope was −0.01 min^–1^ and *r* was 0.045 for the forearm A dataset. The *t-*test and the ICC values for the comparisons are shown in Table 2. The relationship between the Mito22 and Mito6 data when separated into above and below midpoint fit is shown in Figure 5.

**TABLE 2:** Test statistics.

	Bicep			Forearm A		
		
Comparison	Mito22 Mito6	Mito22 Mito5*	Mito6 Mito5*	Mito22 Mito6	Mito22 Mito5*	Mito6 Mito5*
*t*-Test (p value)	0.93	0.18	0.36	0.86	0.97	0.78
ICC	0.959	0.955	0.957	0.954	0.929	0.965

**P*-values were calculated from paired, two tailed *t*-Test from the multiple trial averages of each individual. ICC is interclass correlation coefficients and were generated using a two-way mixed effects model using average values where people effects are random and measures effects are fixed. The ICC values generated refer to within subject comparisons.*

### Four Repeated Mito6 Test

**FIGURE 6:**
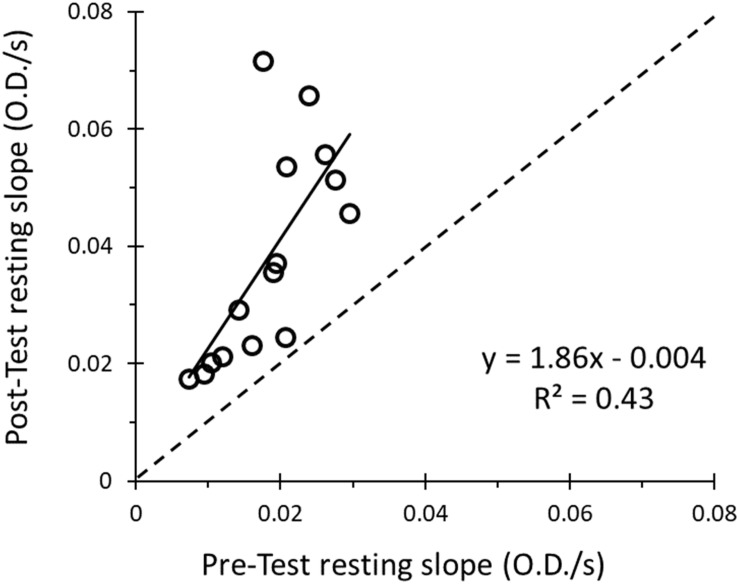
The correlation between the slope of the pre-test resting cuff and the slope of the post-test resting cuff is shown. The resulting equation and *R*^2^-value show a weak positive relationship between pre-test resting cuff value and the post-test resting cuff value (*R*^2^ = 0.43, slope = 1.86).

**FIGURE 7:**
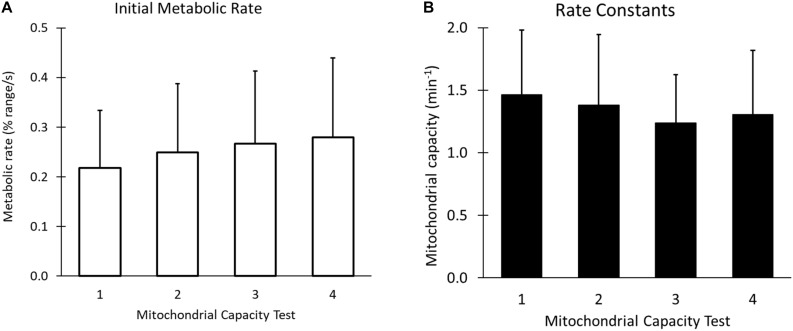
**(A)** The initial metabolic rates, or the slope of the first cuff occlusion in each of the four mitochondrial tests, using the final resting cuff value as the curve endpoint. **(B)** The rate constants of the four tests of a Mito6 protocol. There is no statistical difference between the average of each of the four tests, and no order effect is seen as the Mito6 protocol progresses.

When performing the four 6-occlusion tests; recovery rate constants were not different between tests (1.50 ± 0.51 min^–1^, 1.42 ± 0.54 min^–1^, 1.26 ± 0.41 min^–1^, 1.29 ± 0.47 min^–1^, *P* > 0.05). The final resting value was 1.86 times higher than the initial resting value (*P* < 0.001) (Figure 6). The initial metabolic rates are shown in Figure 7A. The rate constants of four mitochondrial capacity tests are shown in Figure 7B. The results show that as the number of mitochondrial tests increases, the initial rate of metabolism increases (*P* < 0.01). There were no order effects in the Mito6 rate constants seen with repeated testing (*P* > 0.05).

## Discussion

The primary finding of this study was that measured mitochondrial rate constants were equivalent (absence of a meaningful effect) when calculated from a reduced number of data points (Mito6) compared to a full set of data points (Mito22). The conclusions were based on equivalency testing (Lakens, 2017) and Bland–Altman analysis (Altman and Bland, 1983; Dogan, 2018). The results of this study therefore support the validity and repeatability of the Mito6 protocol. Previous studies have measured muscle mitochondrial capacity using exercise to rest transitions and curve fitting similar to the Mito22 used in this study (Willingham and McCully, 2017; Adami and Rossiter, 2018). Collecting data points throughout the entire recovery process can result in accurate curve fits, as indicated by coefficients of variation between 8 and 12% (Ryan et al., 2013a; Southern et al., 2014). However, a limitation of this approach is that a large number (Altman and Bland, 1983; Miura et al., 2001; Sako et al., 2001; Brizendine et al., 2013; Southern et al., 2014; Bossie et al., 2017; Lakens, 2017; Dogan, 2018; Barstow, 2019) of short ischemic periods are needed to perform accurate curve fitting. The Mito6 protocol only requires 7 ischemic periods (A Mito6 test with one additional point for the full recovery time point). Thus, the Mito6 protocol significantly reduces the number of ischemic periods needed, especially if 2 or 3 experiments are performed to increase the accuracy of the measurement (Erickson et al., 2013; Bossie et al., 2017). We are not aware of any previous studies that have used incomplete recovery curves to determine a mitochondrial capacity rate constant.

In addition to requiring fewer ischemic periods, a second benefit of the Mito6 protocol is a reduced reliance on data points with low metabolic rates (which occur later in the recovery process). With continuous wavelength NIRS devices, as used in this study, inflating a blood pressure cuff can change the scattering of light which is not directly detected by the continuous wavelength NIRS device (Barstow, 2019). The changes in scattering appear to be similar in magnitude on the absorption signal to the changes in absorption of resting metabolism (Sako et al., 2001; Ryan et al., 2012). Because of this, corrections for scattering changes (“blood volume correction”) must be very accurate or there will be errors in correcting these data points, which will influence the fitting of the exponential curve. During the early points in recovery, the metabolic rate is much higher, and thus the influence of the blood volume correction factor is far less. The Mito6 protocol thus has an advantage over the Mito22 protocol, as it is not as dependent on accurate corrections for changes in scattering.

This study chose to measure the initial six recovery points to find the rate constant of the exponential curve. This seems appropriate for the rate constants found in the two studies that were evaluated (approximately 1.5 min^–1^). With these rate constants, the first six values occur during the steepest part of the recovery curve where metabolic rate is activated. Studies of endurance trained athletes (Brizendine et al., 2013) or people with reduced mitochondrial capacity (Erickson et al., 2013) may benefit from a different number of initial data points. In our study we did evaluate the potential value of using five initial data points based on reducing the residuals of the curve fit, rather than all six of the initial points. Because removing one data point provided the same equivalence measures, it cannot be determined if removing a point provides meaningful benefits or not. Removing an outlying point has the potential to improve the fit of the data. However, based on the data, there was not a consistent benefit to removing an outlying point. Future studies could evaluate the benefit of excluding a data point in the Mito6 approach.

Almost any method of fitting data should work well when the data was collected with precision. A possible limitation to the use of the Mito6 approach is how well the method would fit data that had lower “quality.” It is expected that all methods of curve fitting, including those proposed here or any other possible method, would work on higher quality data. However, a key differentiation between methods is how they are able to analyze poorly collected data. The determination of what was considered “good quality” and “bad quality” data in this study was decided arbitrarily based on the goodness of fit of the exponential curve. Because we found the six-cuff approach appeared to work well on the bad quality data as well as on the good quality data, it suggest that this approach would obtain accurate results in data sets with varying levels of quality. In general, NIRS based recovery measurements of mitochondrial capacity have better curve fits than ^31^P MRS fits of phosphocreatine recovery after exercise (Ryan et al., 2013b). However, some study populations have greater adipose tissue thickness over the muscle of interest (Erickson et al., 2013), and great adipose tissue can reduce the quality of the data for NIRS studies (Miura et al., 2001). This study evaluated two data sets on relatively young and healthy subjects, one on the biceps muscle and the other on forearm muscles to establish validity of the Mito6 approach. The study also evaluated a proposed Mito6 protocol on a forearm muscle. Additional studies evaluating the Mito6 approach should be done on data sets where there is reduced signal and the quality of the data is less.

Another finding of this study was that the four rate constants determined from performing repeated Mito6 tests were not significantly different from each other. Previous studies have shown that two 22-cuff tests in a row give the same value (Brizendine et al., 2013), and our data agrees with that finding. Completing two 22-cuff tests takes a total of nearly 45 min, and our new six-cuff protocol takes a total of 25 min and produces data from four mitochondrial capacity tests. This suggests that repeating the six-cuff tests can be used to increase accuracy without the limitation of an order effect over time. The advantage of the six-cuff protocol is that the tests are shorter, so more testing and data collection can be done in the same amount of time with less stress placed on participants.

Our study found that the initial resting slope was much smaller than the final resting slope, most likely due to post exercise oxygen consumption. Because of this, a final resting cuff value is critical for the process of curve fitting.

## Conclusion

The Mito6 analysis protocol which uses six data points along with a final recovery time point can be used as an alternative to the currently used Mito22 analysis protocol. Discarding an outlying point did not have a clear benefit. While the newly proposed protocol does require a post exercise resting measurement, it still shortens the testing period. An advantage of the Mito6 approach is that four rate constant measurements can be made in the same time as it takes to perform two traditional complete recovery measurements. Future studies of mitochondrial capacity using NIRS should consider using this approach.

## Data Availability Statement

The raw data supporting the conclusions of this article will be made available by the authors, without undue reservation, to any qualified researcher.

## Ethics Statement

The studies involving human participants were reviewed and approved by the Institutional Review Board – University of Georgia. The patients/participants provided their written informed consent to participate in this study.

## Author Contributions

MS performed the data collection, data analysis, and wrote the first draft of the manuscript. SB performed the data collection, data analysis, and contributed to the writing of the manuscript. EP performed the data collection and contributed to the writing of the manuscript. ID assisted with data analysis and contributed to writing of the manuscript. KM designed the study, contributed to data analysis and interpretation, and contributed to writing of the manuscript.

## Conflict of Interest

One of the authors, KM, is the President and Chief Science Officer of Infrared Rx, Inc., a company that develops analysis software related to the NIRS measurements. The remaining authors declare that the research was conducted in the absence of any commercial or financial relationships that could be construed as a potential conflict of interest.
